# Mammalian CD1 and MR1 genes

**DOI:** 10.1007/s00251-016-0926-x

**Published:** 2016-07-28

**Authors:** Peter Reinink, Ildiko Van Rhijn

**Affiliations:** 1 Division of Rheumatology, Immunology and Allergy, Brigham and Women’s Hospital, Harvard Medical School, Boston, MA USA; 2 Department of Infectious Diseases and Immunology, Faculty of Veterinary Medicine, Utrecht University, Utrecht, The Netherlands

**Keywords:** CD1, Lipid antigens, Mammals, MR1

## Abstract

All higher vertebrates share the fundamental components of the adaptive immune system: the B cell receptor, the T cell receptor, and classical MHC proteins. At a more detailed level, their immune systems vary considerably, especially with respect to the non-polymorphic MHC class I-like proteins. In mammals, the CD1 family of lipid-presenting proteins is encoded by clusters of genes of widely divergent sizes and compositions. Another MHC class I-like protein, MR1, is typically encoded by a single gene that is highly conserved among species. Based on mammalian genomes and the available data on cellular expression profiles and protein structure, we review MR1 genes and families of CD1 genes in modern mammals from a genetic and functional perspective. Understanding the CD1 and MR1 systems across animal species provides insights into the specialized functions of the five types of CD1 proteins and facilitates careful consideration of animal models for human diseases in which immune responses to lipids and bacterial metabolites play a role.

Whereas MHC genes and the genes for T cell receptors appeared simultaneously during evolution and are present in all extant jawed vertebrates, the genes for CD1 and MR1 have a more limited distribution. Keeping the phylogenetic relationships among animal species in mind, we will describe the CD1 and MR1 genes in extant species. The evolution of these genes is beyond the scope of this review. CD1 and MR1 proteins bind lipids and vitamin B metabolites respectively, and present these to T cells, as opposed to the classical MHC proteins, which present peptide antigens to T cells. CD1 and MR1 genes have not been found in fish, while fish do contain MHC class I and II genes (Dascher 2007). Reptiles (Yang et al. 2015), birds (Miller et al. 2005; Salomonsen et al. 2005), and marsupials (Baker and Miller 2007; Cheng and Belov 2014) have CD1 genes that can clearly be distinguished from classical MHC genes and form an interspecies group with the mammalian CD1 genes. CD1a, CD1b, CD1c, CD1d, and CD1e proteins most likely arose in a common ancestor of placental mammals from a primordial form of CD1. Gene duplications, deletions, and gene inactivations shaped the composition of the CD1 family of genes, possibly under selective pressure associated with an immune function. MR1 genes are absent in fish and reptiles, but in marsupials and mammals, there is typically a single functional MR1 gene. Of all the MHC Class I-like proteins, MR1 shows the highest conservation among species.

## Chromosomal location and gene structure of CD1 and MR1

In mammals, the CD1 and MR1 genes are not part of the MHC locus. In humans, the CD1 and MR1 genes are located on chromosome 1 and the MHC locus lies on chromosome 6. However, in chicken, the CD1 and MHC loci are linked (Miller et al. 2005; Salomonsen et al. 2005). The genes that encode the classical MHC molecules are highly polymorphic with hundreds to thousands allelic variants, and this is thought to be closely related to their function of presenting peptides to T cells. No allelic variants for CD1b, CD1c, and MR1 are known. Allelic variants in the form of non-synonymous single nucleotide polymorphisms in human CD1a and CD1d are known, but the affected amino acids are not located in the antigen-binding cleft or the TCR-binding surface (Han et al. 1999; Oteo et al. 2001). Together, this justifies the common description of CD1 and MR1 genes as non-polymorphic.

Because of its non-polymorphic nature, shared structural features, and the fact that it presents small metabolite antigens rather than peptides, MR1 is often compared to CD1. However, MR1 genes are distinct from CD1 genes, and, based on sequence alignments, form their own interspecies group. Even though in humans MR1 and CD1 are located in the same MHC paralogous region on chromosome 1, the MR1 locus is separate from the CD1 cluster of genes, or CD1 locus (Hashimoto et al. 1995; Shiina et al. 2001). The CD1 locus is located between the KIRREL and olfactory receptor genes and consists of multiple genes, often in both orientations. The MR1 gene is embedded between STX6 and IER5 genes. In mice, a chromosomal rearrangement caused the separation of the CD1 and MR1 genes, which are located on chromosome 3 and chromosome 1, respectively (Dascher and Brenner 2003). In the available assembled primate data, MR1 and CD1 are located on the same chromosome, but in other mammals that were studied, like cow and pig, they are not (Goldfinch et al. 2010; Reinink and Van Rhijn 2016).

The human genome encodes one MR1 protein and five different CD1 proteins, called CD1a through CD1e. These five CD1 proteins and their orthologs are called CD1 isoforms. Even though for other proteins this term is used to define RNA splice variants derived from the same gene, in the CD1 field, the word isoforms is used to indicate products of separate genes. The overall structure of CD1 and MR1 proteins resembles MHC class I molecules: a type I transmembrane protein, called the heavy chain, consisting of α1, α2, and α3 domains associated with β2 microglobulin. CD1 and MR1 genes have an intron-exon structure comparable to MHC class I genes: they contain 6 exons that encode 5′ UTR and leader signal peptide, α1 domain, α2 domain, α3 domain, transmembrane domain, and cytoplasmic tail and 3′ UTR combined (Yamaguchi et al. 1998). CD1 genes in mammals are named after the human CD1 isoform they group with, based on sequence comparison. Comparison, based on overall alignment of the full coding sequence or only the α1 and α2 domains that form the antigen binding cleft, gives identical results. This is caused by the fact that α3 domains are highly conserved among all CD1 isoforms within one species (Balk et al. 1989), and cytoplasmic domains, even though they show considerable differences among the isoforms, are very short. Thus, effectively, CD1 isoforms are grouped and named according to resemblance of the sequence encoding their antigen-binding cleft-forming α1 and α2 domains.

## CD1 in humans and common research, farm, and companion animals

For nine mammalian species (human, rabbit, guinea pig, cow, pig, dog, horse, mouse, and rat), the CD1 genes have been studied extensively and, except for rabbit and guinea pig, their CD1 loci have been carefully mapped and curated. The functionality of many of these CD1 genes has been studied by cloning the transcripts from cDNA, sometimes followed by protein expression studies. Genomes from these nine mammalian species contain from one (rat (Katabami et al. 1998)) to thirteen (horse and dog (Dossa et al. 2014; Schjaerff et al. 2016)) CD1 genes. From rabbits, two CD1a, two CD1b, one CD1d, and one CD1e transcripts have been identified, but this study was not set up or intended to define the complete rabbit CD1 locus (Hayes and Knight 2001). Southern blots suggested the presence of at least eight CD1 genes in the rabbit (Calabi et al. 1989). Guinea pigs have been reported to contain four functional genes for CD1b, three for CD1c, one for CD1e gene, and at least five CD1 pseudogenes (Dascher et al. 1999). Later, one functional gene for CD1d was described (Looringh van Beeck et al. 2009), and one gene of unknown functionality for CD1a (Van Rhijn and Moody 2015). The guinea pig CD1 locus has not been mapped, but while the genome is being updated and assembled, even more CD1 genes have been identified in guinea pig (Reinink and Van Rhijn 2016). The first attempt to define the bovine CD1 locus was based on an early draft of the bovine genome and identified one CD1a gene, five CD1b genes, two CD1d genes, and a CD1e gene. Among these, the CD1a gene, three CD1b genes, and the CD1e were identified as functional genes (Van Rhijn et al. 2006). A later version of the genome brought the number of bovine CD1 genes to 12 (Nguyen et al. 2015). The porcine CD1 locus has been described based on BAC sequencing and contains six CD1 genes: two genes for CD1a, and one for each of the other isoforms (Eguchi-Ogawa et al. 2007). One of the CD1a genes is a pseudogene. Twelve CD1 genes were mapped in the canine locus based on the available genome at the time, and a 13th gene was not mapped in the locus and was thought to be an allele (Looringh van Beeck et al. 2008). Subsequently, BAC sequencing placed the 13th gene in the locus, which is now known to contain nine CD1a genes, and one gene or each of the other isoforms (Schjaerff et al. 2016). Four of the canine CD1a genes are thought to be functional. The horse genome contains 18 CD1 genes, among which five pseudogenes (Dossa et al. 2014). The functional equine CD1 genes are seven CD1a genes, two CD1b genes, one CD1c gene, one CD1d gene, and two CD1e genes. The mouse genome, with its small locus consisting of only two CD1d genes, has been shown to have undergone a rearrangement that caused the loss of the other CD1 genes (Bradbury et al. 1988; Dascher and Brenner 2003). Rats have only one CD1 gene, which encodes CD1d (Ichimiya et al. 1994).

## CD1 loci in less well studied mammalian genomes

Only a very small number of CD1 loci of the total of almost 4000 extant species of placental mammals have been studied. For species other than human, rabbit, guinea pig, cow, pig, dog, horse, mouse, and rat, the study of CD1 genes is now facilitated by the availability of multiple genomes. It should be noted however that many genomes, especially from species other than primates and rodents, are not yet finalized and contain multiple gaps. Also, not all sequence materials may have been assigned to a chromosomal location yet, and artifacts like duplications may be present. Therefore, attempts to describe a CD1 locus based on genomic data available at a certain point in time may need to be adjusted later based on improved versions of the genome (Nguyen et al. 2015; Schjaerff et al. 2016). Because transcription and correct splicing of existing genes cannot reliably be predicted, mRNA- and cDNA-based sequences provide the most reliable data on functionality and the possible expression of individual CD1 proteins in vivo. However, RNA sequence-based data are unlikely to provide a complete overview of CD1 proteins in a species because expression of CD1 genes is often limited to a specific tissue and cell type. Therefore, to get an indication of the total number of CD1 genes in a species, searches using the basic local alignment search tool (BLAST) or collection of annotated genes in genomes provide more reliable information than expressed sequence tags or cDNA databases. Table 1 shows numbers of CD1 genes obtained from whole genomes of 15 mammals, including less well studied species like alpaca, dolphin, elephant, two bat species, panda, and sloth (Reinink and Van Rhijn 2016). Of note, BLAST-based searches reveal functional genes and pseudogenes. Therefore, differences between results of de novo BLAST searches, automated annotation of open reading frames, and published data with regard to the total number of CD1 genes per species are expected.

**Table 1:** CD1 and MR1 gene numbers

Common name	Genome	Binomial species name	CD1a	CD1b	CD1c	CD1d	CD1e	Total CD1	MR1
Alpaca	vicPac2	*Vicugna pacos*	1	1	1	1	1	5	1
Bonobo	panPan1	*Pan paniscus*	1	1	1	1	1	5	2
Chimpanzee	panTro4	*Pan troglodytes*	1	1	1	1	1	5	2
Dog	CanFam3	*Canis lupus*	9	1	1	1	1	13	1
Dolphin^a^	turTru2	*Tursiops truncatus*	0	1	0	0	0	1	0
Elephant	loxAfr3	*Loxodonta africana*	1	2	1	1	1	6	1
Horse	equCab2	*Equus caballus*	9	2	2	1	2	16	1
Human	hg38	*Homo sapiens*	1	1	1	1	1	5	2
Megabat	pteVam1	*Pteropus vampyrus*	3	1	1	0	1	6	1
Microbat	myoLuc2	*Myotis lucifugus*	17	2	0	5	2	26	1
Mouse	mm10	*Mus musculus*	0	0	0	2	0	2	1
Panda	ailMel1	*Ailuropoda melanoleuca*	8	1	1	1	1	12	1
Pig	susScr3	*Sus scrofa*	2	1	1	1	2	7	1
Rabbit	oryCun2	*Oryctolagus cuniculus*	5	2	0	1	2	10	0
Rhesus macaque	rheMac3	*Macaca mulatta*	2	1	1	1	1	6	2
Sloth^a^	choHof1	*Choloepus hoffmanni*	1	0	0	1	0	2	1

For each of the indicated mammalian genomes, a list of CD1 and MR1 genes as determined by BLAST-based searches was merged with a list of Ensembl-annotated CD1 and MR1 genes when available (adapted from (Reinink and Van Rhijn 2016)). Redundancies (genes with identical genomic location) were removed

^a^The dolphin and sloth genomes are not completely assembled and consist of relatively small contigs, which may have led to the fragmentation of CD1 or MR1 genes and subsequent failure of identification

**Fig. 1:**
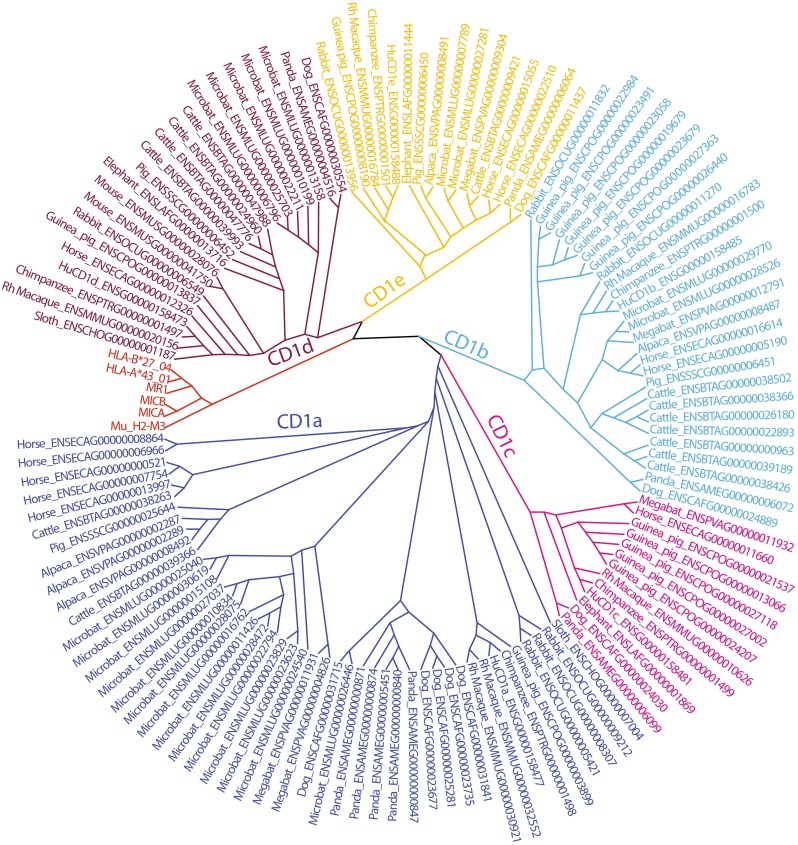
CD1 and MR1 genes in mammals. From 16 mammals, known CD1 genes and predicted CD1 paralog open reading frames were obtained from Ensembl (www.ensembl.org). An alignment of these sequences and human MICA, MICB, HLA-A, HLA-B, and murine H2-M3 was generated by MUSCLE (Edgar 2004), clustered according to a neighbor joining algorithm, and shown as a radial cladogram. Groups were color-coded based on the clustering with human CD1 isoforms

CD1 genes have undergone multiple duplications in most mammals, leading to extended multigene families. The general picture that emerges is that CD1a has undergone the most extensive multiplications and CD1e the least or none. Automated annotations of CD1 isoforms of species other than mouse or human often indicate a certain degree of uncertainty, and are sometimes annotated as “CD1a-like,” for example. However, the golden standard for isoform assignment is sequence alignment of the combined α1 and α2 domains. All CD1 genes from 15 mammals that were recovered group with one of the five known CD1 isoforms, but were not always correctly named by automated annotation (Fig. 1). The clear grouping with the five isoforms suggests that the isoform nomenclature that was based on the human CD1 locus appropriately describes all currently known mammalian CD1 isoforms. With more than 10 CD1 genes, the little brown bat, *Myotis lucifugus*, can be considered a species with high numbers of CD1 genes. Intermediate numbers are found primates, alpaca, and elephant. These data confirm once more that mouse and rat are atypical with the lowest absolute number of CD1 genes (two and one, respectively). Though not identical, the gene numbers obtained by BLAST-based searches and automated annotation are largely consistent (Reinink and Van Rhijn 2016). Because misassemblies of repeated sequences and unmerged overlaps due to polymorphisms resulting in artificial duplications can occur in incompletely assembled genomes, we expect that in some species, the exact numbers of CD1 genes will be adjusted in the future. However, it is clear that humans should be considered to have an intermediate number of CD1 genes, while many other mammals have more extensive families of CD1 genes. Large CD1 families are often dominated by multiplied CD1a genes and to a lesser degree, CD1b, CD1c, and CD1d genes.

## Mammalian MR1 genes

Searches in whole genomes (Table 1), as well as other published data, show that primates have two MR1 genes. In humans and chimpanzees, one of these genes is known to be functional and the other one is a pseudogene (Parra-Cuadrado et al. 2001; Parra-Cuadrado et al. 2000). No MR1 gene could be identified in the rabbit genome, which is in line with recently published data on lagomorphs (rabbit and pika) (Boudinot et al. 2016). The panda and dog MR1 genes that resulted from BLAST-based searches (Table 1) are most likely pseudogenes because it was shown that members of the order of Carnivora, including cats, dogs, ferrets, and pandas, have a MR1 pseudogene and lack a functional MR1 gene (Boudinot et al. 2016). Other mammals (alpaca, elephant, horse, bat) also have one MR1 gene. While in dolphin no MR1 gene was found, this does not conclusively prove the absence of MR1 in this species because the dolphin genome is still incompletely assembled. The sloth MR1 gene has a gap in the current version of the sloth genome (choHof1).

## Functional specialization of CD1 isoforms

Interspecies comparisons between individual CD1 proteins have been made based on sequence alignment, protein models (canine versus human CD1a (Looringh van Beeck et al. 2008)), or crystal structures (bovine versus human CD1b (Girardi et al. 2010), bovine versus human CD1d (Wang et al. 2012), and murine versus human CD1d (Koch et al. 2005)). CD1 proteins within one species differ from each other in ways that suggest functional specialization. One aspect determines the function of a CD1 protein is the size and shape of the antigen-binding cleft, which is formed by the α1 and α2 helices. Among the human CD1 proteins, the biggest size difference is observed between CD1a and CD1b. In addition, there are significant differences in shape with the CD1b cleft consisting of four pockets (Gadola et al. 2002) of which three are interconnected and the CD1a cleft consisting of one cavity (Zajonc et al. 2003). These differences translate into differences in size and shape of antigens that can be bound by the human CD1a and CD1b proteins. Because the CD1 isoforms form interspecies groups based on phylogenetic relationship based on the sequences of their α1 and α2 helices, functional specializations of CD1 molecules that relate to the antigen-binding cleft are generally conserved across species. Especially, the size of the cleft seems to be well conserved when human CD1 isoforms are compared with an ortholog. However, as seen in the bovine CD1d molecule, despite a very high homology between human and bovine CD1d, tryptophan 166 in the bovine protein, which is a cysteine in humans and mice, blocks part of the A’ pocket so that long fatty acyl chains will not fit (Wang et al. 2012). A comparable situation exists when human CD1b and bovine CD1b3 are compared: the tunnel that is present in the human CD1b protein is closed in bovine CD1b3 by valine 98, which prevents the binding of extremely long ligands (Girardi et al. 2010). Structural aspects of these CD1 molecules will be reviewed by Zajonc et al. in this special issue of Immunogenetics. Thus, despite very high sequence homology and overall structural resemblance between orthologs, a single amino acid difference can have profound functional impact.

One specific feature of CD1 molecules that is difficult to predict from crude genomic data is the cytoplasmic tail, which determines the transport and subcellular location of individual CD1 isoforms (Moody and Porcelli 2003). The cytoplasmic tail is encoded by the very small exon 5 that is located between two longer introns of variable length and cannot reliably be predicted based on the genomic sequence of the CD1 gene. However, the many cDNA sequences that are available have provided a number of cytoplasmic tails that allows for comparative analysis and predictions on intracellular trafficking of these CD1 proteins (Table 2).

**Table 2:** Cytoplasmic tails of CD1 proteins in mammals

Gene name (alias)	Cytoplasmic tail	Motif	Reference cDNA
boCD1a2	RKSWSTYMSDA		(Nguyen et al. 2015)
boCD1a1	WKHWTHRESPSSVLPLE		(Van Rhijn et al. 2006)
boCD1a			
canCD1a2	KAHWRPQCMDFPSEREPSSPSSSTYLNPAQH		(Schjaerff et al. 2016)
canCD1a6	KRWKTHNRPQCTDFPLK		(Looringh van Beeck et al. 2008)
canCD1a9	KAHWRPQCTDFPSEQEPSSPGSSTYLNPAQH		(Looringh van Beeck et al. 2008; Schjaerff et al. 2016)
canCD1a8.1			
canCD1a8	KRWKAH		(Looringh van Beeck et al. 2008; Schjaerff et al. 2016)
canCD1a8.2			
eqCD1a1	THCEAPCTIVPLK		(Dossa et al. 2014)
eqCD1a2	IRHQLQRTLLPLD	Dileucine	(Dossa et al. 2014)
eqCD1a3	IHSELPRTLLPLE	Dileucine	(Dossa et al. 2014)
eqCD1a4	VISISVSILVRKPCATPRTPLPSQ		(Dossa et al. 2014)
eqCD1a5	RSCESASNLLWNEIPGAQDPGHI	Dileucine	(Dossa et al. 2014)
eqCD1a6	WLRKRWTRCEPPSNLISLE		(Dossa et al. 2014)
eqCD1a7	WLRKRGTHCEFPRTCLPLE		(Dossa et al. 2014)
huCD1a	RKRCFC		(Calabi and Milstein 1986; Martin et al. 1987)
pigCD1a1	WHRKHWKHCDPSSALHRLE		(Chun et al. 1999; Eguchi-Ogawa et al. 2007)
pCD1.1			
rabCD1a1	RKCWIHHGPLETLLPLQ	Dileucine	(Hayes and Knight 2001)
rabCD1a2	KKRWSHHGSPNSLLPLK	Dileucine	(Hayes and Knight 2001)
boCD1b1	RFMGSHRVGHD		(Nguyen et al. 2015; Van Rhijn et al. 2006)
boCD1b3	RRWSYQNIL	YXXZ, dileucine	(Nguyen et al. 2015; Van Rhijn et al. 2006)
boCD1b5	RRWSYQTIL	YXXZ, dileucine	(Nguyen et al. 2015)
canCD1b	RRWSYQSIS	YXXZ	(Looringh van Beeck et al. 2008)
eqCD1b1	SYQNIS	YXXZ	(Dossa et al. 2014)
eqCD1b2	SYLNIP	YXXZ	(Dossa et al. 2014)
gpCD1b1	RRWSYEDIL	YXXZ, dileucine	(Dascher et al. 1999; Hiromatsu et al. 2002)
gpCD1b2	KHWSYQDIL	YXXZ, dileucine	(Dascher et al. 1999; Dascher et al. 2002)
gpCD1b3	RRLRCEGIF		(Dascher et al. 1999; Dascher et al. 2002)
gpCD1b4	RRWSYEDIF	YXXZ	(Dascher et al. 1999)
huCD1b	RRRSYQNIP	YXXZ	(Martin et al. 1987)
ovCD1b	RRWSYQNIL	YXXZ, dileucine	(Ferguson et al. 1996)
scd1b42			
ovCD1b	RRWSHRNIL	Dileucine	(Ferguson et al. 1996)
scd1b52			
ovCD1b	RRWSYLTIL	YXXZ, dileucine	(Ferguson et al. 1996)
scd1a25			
pigCD1b	RRWSYQSVL	YXXZ	(Eguchi-Ogawa et al. 2007)
rabCD1b	RRRSYQNIL	YXXZ, dileucine	(Calabi et al. 1989; Hayes and Knight 2001)
canCD1c	RKCCSYQDIP	YXXZ	(Looringh van Beeck et al. 2008)
eqCD1c	SYQNIQRDSSPCFPHCNENCTAQEHRTTE	YXXZ	(Dossa et al. 2014)
gpCD1c1	KRCTYQGIQ	YXXZ	(Dascher et al. 1999)
gpCD1c2	KRCTYQGIP	YXXZ	(Dascher et al. 1999)
gpCD1c3	KKCCTYQGIP^a^	YXXZ	(Dascher et al. 1999)
huCD1c	KKHCSYQDIL	YXXZ, dileucine	(Martin et al. 1987)
eqCD1d	KKRSSYQDIL	YXXZ, dileucine	(Dossa et al. 2014; Looringh van Beeck et al. 2009)
gpcD1d	RRGRSYQDIL	YXXZ, dileucine	(Looringh van Beeck et al. 2009)
huCD1d	RFKQTSYQGVL	YXXZ, dileucine	(Balk et al. 1989)
lafCD1d	KRHCS		(Looringh van Beeck et al. 2009)
moCD1d1	RRRSAYQDIR	YXXZ	(Bradbury et al. 1988)
moCD1d2	RRRSAYQDIR	YXXZ	(Bradbury et al. 1988)
ovCD1d	RKHRRYQDIS	YXXZ, dileucine	(Rhind et al. 1999)
scd1d			
pigCD1d	RRRVYQNIQ	YXXZ	(Eguchi-Ogawa et al. 2007)
rabCD1d	RRRCSYQGIL	YXXZ, dileucine	(Calabi et al. 1989; Looringh van Beeck et al. 2009)
ratCD1d	RRRSYQDIM	YXXZ	(Ichimiya et al. 1994)

The cytoplasmic tails of CD1 proteins, grouped by isoform. Only tail sequences that have been confirmed by cDNA sequences are included. Species from which the sequences were derived are indicated as bo: bovine; can: canine; eq: equine; gp: guinea pig; hu: human; laf: African elephant; mo: mouse; ov: ovine; rab: rabbit; dileucine: modified dileucine motif

^a^The sequence shown is based on GenBank sequence NM_001172855, but has been published as KKCCTYQGFP (Dascher et al. 1999)

## Intracellular trafficking is determined by the CD1 cytoplasmic tail

Cellular lipids are bound in the antigen binding cleft during synthesis of the CD1 protein. When the CD1 protein recycles and encounters other lipids in an endosomal compartment, these may replace the initial endoplasmic reticulum-derived lipid. The type of endosomal compartment a lipid travels to is thus a determining factor in the kind of antigenic lipids it presents. The cytoplasmic tails of CD1 molecules may or may not contain short consensus sequences for adaptor proteins. CD1b, CD1c, or CD1d molecules often carry a modified dileucine motif and/or a tyrosine-based YXXZ motif, where X is any amino acid, and Z is a bulky hydrophobic amino acid. YXXZ motifs interact with adaptor proteins and are responsible for recycling from the cell surface to intermediate and late endosomal compartments (Briken et al. 2002; Sugita et al. 2002). CD1e does not appear at the cell surface and does not recycle and has a function in lipid antigen loading and processing (Angenieux et al. 2005; de la Salle et al. 2005).

Comparing the cytoplasmic tails of all cell surface-expressed isoforms across species, the following general picture emerges: CD1a tails are highly variable in length. Most CD1a tails contain histidine residues, with unknown function, or cysteines, which can be palmitoylated. The human CD1a cytoplasmic tail contains a cysteine residue that is palmitoylated and involved in incorporation in lipid rafts (Barral et al. 2008; Sloma et al. 2008), but the mechanism of recycling to the sorting or early endosomal compartment, which is so typical for human CD1a, is unknown. None of the 17 known cytoplasmic tails of CD1a proteins has a modified dileucine- and/or a tyrosine-based YXXZ motif, consistent with the absence from intermediate and late endosomes of the human CD1a protein, and strongly suggesting that this is an evolutionary conserved feature (Table 2 ). CD1b, CD1c, and CD1d tails are of comparable length, typically contain a YXXZ motif, and sometimes an additional modified dileucine motif. In addition, all known CD1c cytoplasmic tails contain a cysteine of unknown function. There are exceptions to these general observations, like the bovine CD1b1 (Nguyen et al. 2015), guinea pig CD1b3 (Dascher et al. 1999), and African elephant CD1d (Looringh van Beeck et al. 2009) cytoplasmic tails, which lack any known motif. Indeed, guinea pig CD1b3 does not travel to late endosomes and rather has a CD1a-like subcellular distribution pattern (Dascher et al. 2002). However, the bovine CD1b3 and CD1b5 and guinea pig CD1b1, CD1b2, and CD1b4 have “typical” CD1b cytoplasmic tails. Thus, cows and guinea pigs have typical and atypical cytoplasmic tails among the proteins belonging to the CD1b isoform. For the African elephant, no other cytoplasmic tails have been sequenced from cDNA yet. Generally speaking, it seems that when there are multiple proteins of one CD1 isoform in a species, there is functional diversification, but when a species has just one gene for a certain CD1 isoform, this one gene usually shows the “typical” combination of cleft and tail.

## Expression patterns of CD1 isoforms

Human cortical thymocytes express high levels of CD1a, CD1b, CD1c, and CD1d on their cell surface. Broad CD1 expression in the thymus was confirmed in all other species where CD1 expression was studied (Hiromatsu et al. 2002; Howard et al. 1993; Looringh van Beeck et al. 2008; Van Rhijn et al. 2006). Outside the thymus, the general picture that emerges is that CD1d is widely expressed at a low level, with some specific high CD1d-expressing cells in certain tissues, like hepatic stellate cells in the liver (Winau et al. 2007), and marginal zone B cells in the spleen (Makowska et al. 1999). In humans, CD1b expression is limited to dendritic cells; CD1a is expressed by dendritic cells and at high levels by Langerhans cells in the skin, and CD1c is expressed by dendritic cells and subsets of B cells. Like humans, dogs express CD1a in the skin and thymus (Looringh van Beeck et al. 2008). However, only two of the three studied canine CD1a genes (out of the nine CD1a genes in the dog CD1 locus) were preferentially expressed in the skin. In rabbits, one of the two CD1a genes is preferentially expressed in the skin, while the other one has a more general expression pattern (Hayes and Knight 2001). In guinea pigs, different CD1b genes have different expression patterns in peripheral blood and spleen (Hiromatsu et al. 2002). Mice that are transgenic for the part of the human locus that encodes CD1a, CD1b, CD1c, and CD1e with their endogenous promoters show a CD1 expression pattern that is surprisingly comparable to the human CD1 expression pattern in that all isoforms are expressed on thymocytes and lymph node dendritic cells, while CD1a stands out as highly expressed on Langerhans cells, and CD1c is expressed on B cells (Felio et al. 2009).

## Closing remarks

A typical pattern of gene distribution has been described for immune function-related genes including genes that encode TCRs, immunoglobulins, classical MHC molecules, and NK receptors. This pattern is the result of gene family expansion, diversification, and contraction or “birth and death” evolution, and usually leads to families of genes that include a considerable number of pseudogenes (Nei and Rooney 2005). Furthermore, complete loss of certain family members and expansion of other family members are observed when contemporary animal species are compared. With its many duplications, deletions, and pseudogenes, the CD1 loci seem to follow this pattern. Although MR1 genes have been subject to inactivation in the carnivores, extensive gene family expansion and diversification were not observed in any of the species studied. The lack of functional diversification of MR1 genes is hard to explain but may be related to its seemingly exclusive interaction with the highly specialized MAIT cells.

Even though mammals vary widely in the numbers of CD1 genes they have, some general observations can be made and help understand the function of the different CD1 isoforms. The skin seems to be a preferred site for CD1a expression across species. While CD1a genes have undergone extensive multiplication in some species, even more so than CD1b and CD1c, none of the currently described CD1a proteins contains any known signal for endosomal location. Multiplication and diversification of genes may have occurred in response to changes in the environment, including pathogenic and non-pathogenic microbes, to which evolving mammals were exposed.
